# Estimation of Vitamin C Intake Requirements Based on Body Weight: Implications for Obesity

**DOI:** 10.3390/nu14071460

**Published:** 2022-03-31

**Authors:** Anitra C. Carr, Gladys Block, Jens Lykkesfeldt

**Affiliations:** 1 Nutrition in Medicine Research Group, Department of Pathology and Biomedical Science, University of Otago, Christchurch 8011, New Zealand; 2 School of Public Health, University of California, Berkeley, Berkeley, CA 94720-7360, USA; gblock@berkeley.edu; 3 Faculty of Health & Medical Sciences, University of Copenhagen, DK-1870 Frederiksberg C, Denmark; jopl@sund.ku.dk

**Keywords:** vitamin C, ascorbate, obesity, body weight, vitamin C intake, plasma ascorbate concentrations, vitamin C requirements, dietary vitamin C

## Abstract

Higher body weight is known to negatively impact plasma vitamin C status. However, despite this well-documented inverse association, recommendations on daily vitamin C intakes by health authorities worldwide do not include particular reference values for people of higher body weight. This suggests that people of higher body weight and people with obesity may be receiving insufficient vitamin C in spite of ingesting the amounts recommended by their health authorities. The current preliminary investigation sought to estimate how much additional vitamin C people with higher body weights would need to consume in order to attain a comparable vitamin C status to that of a lower weight person consuming an average Western vitamin C intake. Data from two published vitamin C dose-concentration studies were used to generate the relationship: a detailed pharmacokinetic study with seven healthy non-smoking men and a multiple depletion–repletion study with 68 healthy non-smoking men of varying body weights. Our estimates suggest that an additional intake of 10 mg vitamin C/day is required for every 10 kg increase in body weight to attain a comparable plasma concentration to a 60 kg individual with a vitamin C intake of ~110 mg/day, which is the daily intake recommended by the European Food Safety Authority (EFSA). Thus, individuals weighing e.g., 80 and 90 kg will need to consume ~130 and 140 mg vitamin C/day, respectively. People with obesity will likely need even higher vitamin C intakes. As poor vitamin C status is associated with increased risk of several chronic diseases including cardiovascular disease, these findings may have important public health implications. As such, dose-finding studies are required to determine optimal vitamin C intakes for overweight and obese people.

## 1. Introduction

Higher body weight is known to negatively impact vitamin C status, with an inverse association observed between body weight and plasma ascorbate concentrations reported in numerous epidemiological studies [1,2]. Several factors may contribute to this relationship. Firstly, increased body weight correlates with an increased volume of distribution, which from a purely volumetric perspective results in a lower plasma concentration from the same intake with increased body weight [3,4]. In fact, this relationship constitutes the basis of the lower recommended daily intake for women compared to men in some countries [5]. Secondly, obesity per se has been associated with adipocyte dysregulation and systemic low-grade inflammation presumably resulting in increased oxidative stress and potentially an increased turnover of vitamin C [6,7]. The volumetric dilution premise is supported by a well-conducted depletion–repletion study carried out by Block et al. [8]. This study showed decreasing vitamin C status with increasing body weight in participants receiving an identical dietary intake of vitamin C. We have also previously shown that the lack of response to low dose vitamin C supplementation in participants with hypovitaminosis C was partly due to body weight [9].

Current global vitamin C intake recommendations for adults are typically based on a ‘one size fits all’ paradigm [5]. However, with increasing obesity rates becoming a global pandemic [10], the negative impact of higher body weight on vitamin C status should be taken into consideration in public health recommendations. Furthermore, obesity is a major risk factor for severe cardiometabolic diseases such as type 2 diabetes and cardiovascular disease [11]. These diseases have also been associated with depleted vitamin C status, although body weight per se also appears to be a major predictor of vitamin C status in these conditions [12,13].

Vitamin C has numerous important functions in vivo through acting as a cofactor for a family of biosynthetic and regulatory metalloenzymes, including hydroxylating enzymes involved in gene transcription regulation and epigenetic modifications [14,15]. Of note, optimal kinetics of these vitamin C-dependent enzymes are dependent on adequate in vivo vitamin C concentrations [15,16]. Thus, the decreased vitamin C concentrations observed in people with overweight and obesity may compromise in vivo enzyme functions and thereby increase the already elevated disease risk even further. 

In this preliminary investigation, dose-concentration data from two vitamin C supplementation studies, the Block depletion-repletion study [8], and the Levine pharmacokinetic study [17], were used to estimate how much additional vitamin C intake may be required for people of higher body weight to attain a vitamin C status comparable to that of a lower weight individual ingesting an adequate amount of vitamin C.

## 2. Materials and Methods

The analyses were based on vitamin C dose-concentration data from two studies: Block et al. [8] and Levine et al. [17]. The Block study [8] comprised 68 healthy men who underwent two vitamin C depletion and repletion phases. During the one-month depletion phases they consumed a controlled diet providing 9 mg/day of vitamin C, and each one-month repletion phase provided an additional 108 mg/day of vitamin C (i.e., a total of 117 mg vitamin C/day), from either supplement, fruit, or vegetables. The source of vitamin C did not affect the plasma ascorbate concentrations obtained [18]. The participants did not smoke, drink alcohol, or take aspirin during the study, and consumed only the food and drink provided. Participants were weighed weekly, and caloric intake was adjusted to prevent weight gain or loss during the study. Data from the second repletion phase was used for the current investigation as it was the better fit to the Levine pharmacokinetic data [17]. The final regression model for the second repletion phase was as follows: AUC = −168.5 + 7.6 × AA + 95.7 × dose, R^2^ = 0.55, where AUC is the area under the plasma ascorbate repletion curve (for phase 2); AA is the plasma ascorbic acid values at the beginning of the second repletion cycle; and dose is the vitamin C intake per kg of body weight [8].

The Levine study [17] comprised seven healthy men who underwent a depletion and dose response repletion study over 4–6 months in a hospital metabolic unit. Exclusion criteria were cigarette smoking, use of regular medications, history of kidney stones, glucose-6-phosphate dehydrogenase deficiency, diabetes mellitus, bleeding disorders, or family history of iron overload/hemochromatosis. During depletion they consumed a diet of <5 mg/d vitamin C. Steady-state plasma concentrations were determined at sequential vitamin C doses of 30, 60, 100, 200, 400, 1000, and 2500 mg/day. For the current investigation GraphPad Prism 9 (GraphPad Software, San Diego, CA, USA) was used to fit a variable slope (four parameters) curve to the steady-state plasma ascorbate concentrations as a function of dose. This curve displayed sigmoidal kinetics: AA = 7.4 + (dose^4.3^) × 61.5/(dose^4.3^ + 109 × 10^6^), where AA is the ascorbic acid concentration and dose is the vitamin C intake. This was used to estimate dose (mg/day) based on predicted plasma concentrations (µmol/L), as well as estimate plasma ascorbate concentrations (µmol/L) based on dose (mg/day). 

## 3. Results

**Figure 1:**
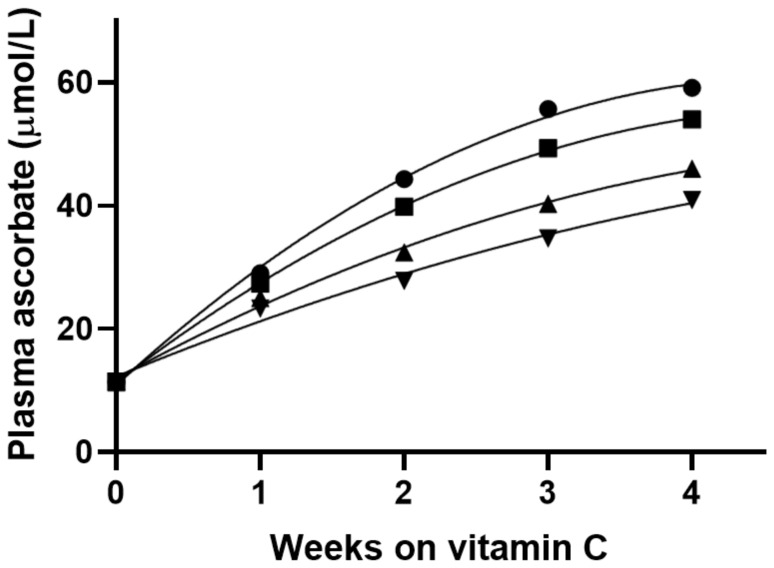
Predicted plasma ascorbate concentration after repletion with vitamin C (117 mg/day) by body weight. The body weights used in the model were: 59 kg (130 lbs; ●), 68 kg (150 lbs; ■), 82 kg (180 lbs; ▲), 91 kg (200 lbs; ▼). The model assumed that all participants started at the same depleted level. Data were obtained from Table 3 in reference [8].

The Block study [8] quantified the effect of body weight on plasma ascorbate concentrations attained on a given dose of vitamin C in 68 healthy non-smoking men of mean age 41 years (range 30–59 years) and mean weight 81 kg (range 59–101 kg). The mean plasma ascorbate concentration attained following depletion was 13 ± 2 µmol/L, and repletion with 117 mg/day vitamin C provided a mean plasma concentration of 49 ± 14 µmol/L. Based on the regression model derived from the plasma ascorbate values following supplementation, the predicted plasma ascorbate concentrations that would be attained after various time intervals in men with various body weights (e.g., 59, 68, 82, and 91 kg) were calculated (Figure 1) [8]. These resulted in predicted plasma ascorbate concentrations of 59, 54, 46, and 41 µmol/L after 4 weeks of supplementation. The difference in plasma ascorbate concentrations between the lowest and highest body weight categories was 18 µmol/L.

**Figure 2:**
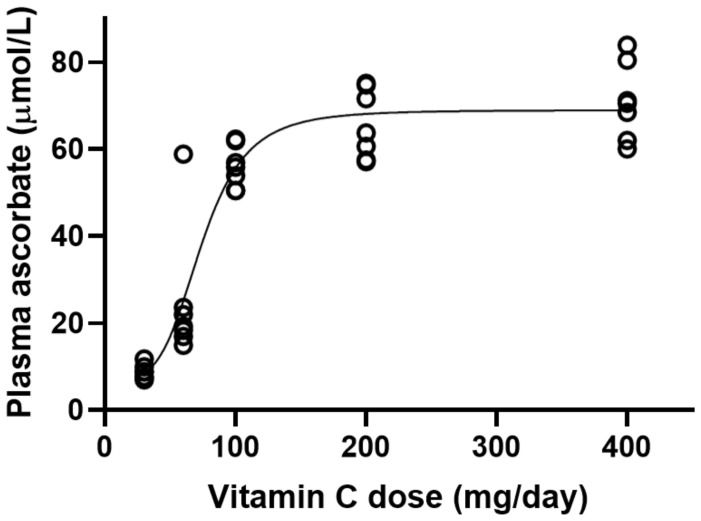
Steady-state plasma ascorbate concentrations in volunteers as a function of daily dose. Data were obtained from Table 1 in reference [17] and a sigmoidal curve was fitted to the data.

For the current investigation, the sigmoidal vitamin C dose-concentration curve from the Levine pharmacokinetic study [17] was used to estimate the vitamin C dose required to obtain the predicted plasma ascorbate concentrations derived in the Block study (Figure 2). This resulted in estimated doses of 109, 97, 84, and 77 mg/day (to obtain the predicted plasma concentrations of 59, 54, 46, and 41 µmol/L, respectively). The 117 mg/day dose administered in the Block study equated to an estimated 61 µmol/L plasma ascorbate concentration using the Levine curve, i.e., a mere 2 µmol/L more than the plasma concentration predicted in the Block study for the lowest body weight (i.e., 59 µmol/L for 59 kg person), suggesting that the predicted data for the low body weight category in the Block study was an excellent fit to the Levine dose-concentration data.

**Figure 3:**
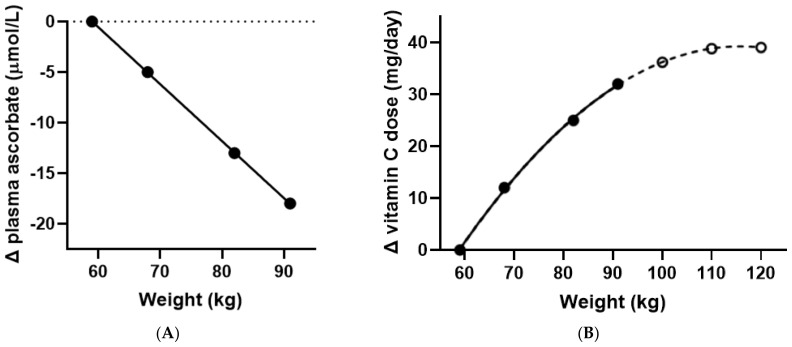
Decreased steady-state ascorbate concentrations with weight (**A**) and increased vitamin C requirements with weight (**B**). The dashed line represents extrapolation of the weight data points.

The difference in estimated vitamin C doses between the lowest and highest predicted plasma concentrations was 32 mg/day (Table 1). Thus, the data suggest that a 91 kg person would need to consume an extra 32 mg of vitamin C to reach the same plasma ascorbate concentration as a 59 kg person consuming 109 mg, i.e., a total of 140 mg/day. A linear relationship was observed between weight and change in plasma concentration resulting from a 117 mg/d daily intake (Figure 3A). However, when comparing the increase in dose required with increasing weight to attain a plasma concentration similar to a 59 kg individual, a non-linear relationship was observed, particularly when extrapolated to higher body weights (Figure 3B).

**Table 1:** Estimated vitamin C doses based on body weight categories.

	Body Weight Category, kg (lbs)			
	59 (130)	68 (150)	82 (180)	91 (200)
Dose (mg)/kg body weight ^1^	2.0	1.7	1.4	1.3
Predicted plasma ascorbate (µmol/L) ^2^	59	54	46	41
△ Plasma ascorbate (µmol/L)	0	−5	−13	−18
Estimated vitamin C dose (mg) ^3^	109	97	84	77
△ Vitamin C dose (mg)	0	12	25	32
Total vitamin C dose	109	121	134	141

^1^ Supplemented with 117 mg/day [8]; ^2^ After 4 weeks of vitamin C supplementation [8]; ^3^ Vitamin C dose was estimated using the curve from Figure 2 above.

## 4. Discussion

This investigation, drawing on previously published data [8], shows a considerable body weight dependency when translating the same daily intake of vitamin C to the corresponding steady-state plasma ascorbate concentration. Our estimates suggest that within a weight range of approximately 60–90 kg, an additional 10 mg/day vitamin C intake is required for each 10 kg increase in body weight to attain a comparable plasma concentration to that of a 60 kg individual ingesting 110 mg vitamin C/day. This corresponds to 140 mg/day for a 90 kg person, which was the highest body weight data available for this study. However, extrapolating the relationship between body weight and dose beyond 90 kg suggested that the 10 mg/10 kg increase in requirement tapers off when approaching 120 kg body weight. This is probably due to fat mass increasing with increased obesity. Consequently, the volume of distribution for ascorbate, being a highly water-soluble molecule, is unlikely to increase proportionally to weight gain over the full range of obesity. Thus, correcting exclusively for volumetric dilution of vitamin C may not be appropriate for weights over about 110 kg.

However, the estimated 40 mg/day additional requirement for those weighing 110+ kg (Figure 3B) is likely an underestimate because people with obesity will likely need even higher vitamin C intakes for other reasons. As obesity and fat mass increase, so does low grade inflammation mediated by adipokines and oxidative stress [7]. As inflammation and oxidative stress conditions are generally recognised to increase vitamin C turnover, a further increase in vitamin C intake would be required to compensate for obesity. However, the present study does not allow for conclusions regarding vitamin C requirements in people with obesity, as the weight range was too limited.

The present findings have implications for public health policy and nutrition recommendations. A vitamin C intake of 110 mg/day is the recommended dietary intake of the European Food Safety Authority (EFSA) and other European countries (e.g., France, Germany, Austria, and Switzerland) [5]. This intake is to attain a plasma ascorbate concentration of 50 µmol/L, which is considered adequate [19]. Of note, even with a slightly higher intake (i.e., 117 mg/day), participants in the Block study with body weights >80 kg had predicted plasma vitamin C concentrations <50 µmol/L [8]. Many countries globally have recommended vitamin C intakes well below 110 mg/day, some as low as 40 mg/day [5], which provide inadequate plasma concentrations [17], even in people of lower body weight, such as 59 kg [20].

According to the World Health Organisation, more than 1.9 billion (39%) of adults were overweight in 2016 and of these over 650 million (13%) were obese [21]. In some regions of the world, these percentages are considerably higher, e.g., 61% in the Americas, 55% in Europe, and 46% in the Eastern Mediterranean [11]. Furthermore, obesity prevalence of >50% is particularly prevalent in specific genders in a number of low-middle income countries [22]. In 2005, the average global body weight was estimated to be 62 kg, however, the average weight in North America was estimated to be 81 kg [23]. Currently, the average body weight in the USA for adult males is 91 kg [24], with 74% of US adults being overweight, and of these 42% are obese [25]. Therefore, future studies are needed to determine the vitamin C requirements for overweight and obese people in particular.

Other than body weight, the most significant contributors to attainable vitamin C status among non-smokers appear to be baseline vitamin C status and how long the person has been depleted [8]. Such depletion is common in low-income populations and is also a result of chronic diseases or frequent acute illnesses, such as respiratory infections [1]. Smoking is another important factor that results in depletion of vitamin C status due to elevated oxidative stress [5]. These factors will negatively impact not only the attainable vitamin C status, but also the length of time to reach steady-state levels. Of note, in the Block study [8], overweight non-smoking participants did not appear to have reached steady-state values even after one month of daily supplementation with 117 mg/day. Other potential confounders include differences in total body water (which also correlates with total body fat and fat-free mass) between men and women and also between people of different ethnicities [26], suggesting there could be ethnic differences in vitamin C requirements based on volumetric effects. As such, even within the same weight category, there is likely to be significant variation in vitamin C requirements due to the presence of various confounders.

There are several limitations of this preliminary investigation, including the assumption that body weight was the sole contributor to the differences in plasma vitamin C status in the Block study data, as some of the factors mentioned above may have had an impact on the predicted plasma concentrations in the Block study, although the investigators did rule out any major impact from environmental smoke exposure, physical and emotional stress, minor illnesses, and physical activity [8]. As mentioned above, the higher weight participants may not have reached steady-state values, however, the participants also had not been depleted for as long as those in the Levine pharmacokinetic study [17]. The Levine study did not report the ages and weights of the participants included in the study, although they were stated to be healthy non-smoking men [17]. Therefore, the equivalency of the data from these participants to the low body weight category data generated in the Block study is uncertain. Finally, the data used for this investigation is derived from only one dose of vitamin C (117 mg/day). Although this is a highly relevant dose and close to the daily intake recommended by EFSA, other doses may offer different vitamin C requirement profiles based on where the resultant plasma ascorbate concentrations fall on the sigmoidal Levine dose-concentration curve. As such, further dose-finding studies are required to determine optimal vitamin C intakes in overweight and obese people.

## 5. Conclusions

The role of body weight as a significant contributor to attained steady-state plasma ascorbate concentrations should be considered in making public health recommendations. Because recommended vitamin C intakes expressed as mg/day will result in lower plasma ascorbate concentrations in higher weight relative to lower weight people, more than 20 years ago Block et al. [8] indicated that dietary recommendations should be based on a dose per kg body weight, or in terms of a desirable plasma concentration. Alternatively, targeted recommendations are needed for people with higher body weight. It is noteworthy that authorities of many countries have already issued special increased recommended daily intakes of vitamin C for subpopulations with increased risk of vitamin C deficiency such as smokers and pregnant and lactating women [5]. 

The results from the current investigation suggest that to attain comparable plasma concentrations to a 60 kg person consuming an average vitamin C intake of 110 mg/day, i.e., the daily intake recommended by most European countries, an additional 10 mg/day of vitamin C would be required for each additional 10 kg increase in body weight. As such, higher weight people (e.g., 80–90 kg) will require estimated vitamin C intakes of 130–140 mg/day. Because vitamin C uptake is comparable between synthetic and food sources of the vitamin [27], any advice around obtaining vitamin C through food sources should include recommendations for higher numbers of servings of fruits and vegetables for overweight and obese people.

## Data Availability

All available data is reported in the paper.
